# Food Loss and Waste Actions: Experiences of the Costa Rican Food Loss and Waste Reduction Network

**DOI:** 10.3390/foods10102358

**Published:** 2021-10-03

**Authors:** Carolina Bolaños-Palmieri, María Fernanda Jiménez-Morales, Julián Rojas-Vargas, Manrique Arguedas-Camacho, Laura Brenes-Peralta

**Affiliations:** 1 InterAmerican Center for Global Health, Puntarenas 60801, Costa Rica; cbolanos@cisgcr.org; 2 Escuela de Agronegocios, Tecnológico de Costa Rica, Cartago 93549, Costa Rica; labrenes@tec.ac.cr; 3 UNA Campus Sostenible, Vicerrectoría de Administración, Universidad Nacional, Heredia 40302, Costa Rica; julian.rojas.vargas@una.ac.cr; 4 Unidad Acción Ambiental, Escuela de Agricultura de la Región Tropical Húmeda EARTH, Guácimo 70601, Costa Rica; margueda@earth.ac.cr

**Keywords:** food systems, food loss, food waste, multistakeholder

## Abstract

Food Loss and Waste (FLW) reduction and prevention can be crucial entry points to achieve sustainable food systems. However, the complexity of this problem poses the need for multistakeholder and multidimensional approaches. The Costa Rican FLW Reduction Network has been working since 2014 as a collaborative platform that brings together different sectors and disciplines to promote a change through communication and awareness, alliances, and research and innovation. The purpose of our study was to share the experience of Costa Rica in regards to the applied FLW actions and its catalytic effect on FLW innovation. The study was developed through a multimethod approach that included case studies, stakeholder analysis and literacy analysis to provide an overall assessment of the strategy as input for further efforts in this matter. The main findings indicate that collaborative actions among institutions and sectors are vital in promoting FLW reduction; however, FLW innovation is still at an inception phase where financial resources and policy barriers remain as aspects to address. In conclusion, the Costa Rica FLW Network represents an asset to trigger ongoing and future actions, and approaches like an integrated innovation ecosystem must be promoted.

## 1. Introduction

Despite the relevance of food systems in every aspect of human development [1], they face the challenge of providing healthy, affordable, and safe food for a growing population in a stable and environmentally appropriate manner [2]. Modern food supply chains require systemic and integrated approaches to address the complex dynamics among stakeholders, subsectors [3], and stages of the life cycle of food products. Unfortunately, even when food systems are recognized as a critical entry point for many sustainability strategies [4], various food production and consumption patterns are unsustainable. In that regard, Food Losses and Waste (FLW), contained in SDG target 12.3, are considered a symptom of malfunction of the food system [5].

FLW definitions and approaches have been under discussion for over a decade now [6] with different conceptualizations around the world [7,8]. Several publications suggest FLW measuring methods and protocols, and many others portray the causes, implications, and relations of FLW to environmental, economic, and social burdens. Discussions remain active, and recent work can be observed in the International Code Of Conduct (CoC) for FLW [9]. This CoC states that FLW causes are rooted at the micro, meso, and macro levels of food systems; therefore, the causes call for distinct areas of action, alternatives of intervention, and types of innovation to provide solutions. For example, direct or micro-level causes relate to particular productive techniques, inputs, practices or operations, marketing trends, and consumer decisions. In contrast, secondary or meso-level causes include equipment, infrastructure, capacities, organization and linkages among the supply chain actors, excessively stringent quality standards, and confusion due to food date labels. Finally, systemic or macro-level causes create effects in the system that result in micro and meso-level causes, born from institutional, policy, and regulatory frameworks that pose barriers or inadequate support to address the matter [9,10].

Cattaneo et al. (2021) synthesized the hindering factors to advance in FWL reduction. They detected gaps in research and policy to measure and monitor FLW, as well as proper determination of trade-offs, understanding of the cost-benefits of reduction strategies, policies, and interventions, as well as the consideration of the effects of the interactions within food supply chain stages in regards to FLW [11]. Stakeholder collaboration creates benefits to all involved parties; however, structural and sporadic conditions need to be surpassed to successfully trigger permanent and consistent FLW reduction actions [12,13,14,15]. Stakeholder involvement, together with research, knowledge, and innovation, remain key aspects to properly address FLW, whether they create direct solutions (innovations) for the food supply chains or provide evidence for policy-making and systemic approaches [16].

Innovation strategies have transitioned from traditional linear approaches to participatory and collaborative interactions within networks, groups of actors, and stakeholders, often referred to as the innovation ecosystem [17], potentially capturing both the causal and the hindering factors behind actual FLW solutions. An innovation ecosystem is defined as the evolving set of actors, activities, artifacts, institutions, and relations, and the complementary and substitute relations that incide in the innovative performance of a certain context [17]. Consequently, this ecosystem is expected to be shaped by its internal elements, external environment and stakeholders. In this case, FLW innovation ecosystems include different types and levels of innovation (market, product, service, process, disruptive, incremental) [18], which are essential to tackle the macro, meso and micro-level causes of FLW as well as the limitations, opportunities and gaps in such a complex challenge. Different innovation examples to address FLW may include the use of 4.0 industry, equipment and postharvest techniques for extended shelflife, and social interventions that could create alternative value chains, among others [19,20,21].

Regions and countries throughout the world are acting to prevent and reduce FLW at different speeds and ranges. The Latin American and Caribbean (LAC) Region is relevant within FLW scenarios due to its participation in global agri-food supply (14%), trade (23% of global exports), and FLW generation (6% of global FLW) [22]. Paramount efforts allowed LAC to reduce hunger and malnutrition in the decade [23]; however, further action is needed to trigger the still incipient adoption of frameworks for Circular Economy, Bioeconomy, and Food Security and Nutrition that bidirectionally connect to FLW reduction. The LAC Region began to formally address FLW in 2014, motivated by the Regional FAO Office and the creation of a Regional Network of Experts to improve technical capabilities and foster actions according to each country’s context [24].

Costa Rica, one of the 42 LAC countries, is characterized as an upper-middle-income nation with high sustainability indicators, steady economic growth, and one of the lowest poverty indicators in the region [25]. Its agri-food sector is a key job and income generator, allowing for overall food security and exports [26]. Therefore, its interest in more sustainable food systems opened an opportunity to begin addressing FLW through a voluntary network called the Costa Rican Food Loss and Waste Reduction Network (CR-FLW Network, or Red Costarricense para la Disminución de pérdida y desperdicio de alimentos, in Spanish), one of the most active FLW groups in Meso-America. Building on the experiences of the CR-FLW Network during the past years, this study aims to showcase actions in Costa Rica, based on networking and multistakeholder interaction as catalyzers of FLW innovation, describing learned lessons and challenges as input for more effort in this matter.

## 2. Materials and Methods

The Costa Rican FLW strategies and actions are led by the CR-FLW Network, established in November 2014. The group is aligned to the FLW Regional Strategy based on three pillars: communication and awareness, knowledge and innovation, and governance and alliances [24]. The network accounts for 25 active organizations or members and approximately 18 intermittent close stakeholders but non-declared members. It has a non-hierarchical structure where the Coordinator (Tecnológico de Costa Rica, a public university) and the Technical Secretariat (FAO National Office) provide main orientations, facilitate joint projects, and lead discussions. At the same time, participating stakeholders share insights, research outcomes, experiences, capacities, and technical inputs according to their sector. Currently, these participants come from public institutions (government and autonomous), private sector, academia and civil society and even when not institutionalized, the CR-FLW Network has opened spaces to address FLW reduction in the country, including its contribution to the construction of policies that currently undertake this matter into their areas of action.

The outcomes of the CR-FLW Network are analyzed in this paper through a multi-method framework that combines stakeholder mapping, a case study, and a literacy analysis in regards to the enablement of FLW actions and innovations in the country. Altogether, this followed framework aids in the discussion of:

(a) the value of stakeholder collaboration to further FLW actions,
(b) the advantages of stakeholder involvement in the innovation ecosystem to reduce FLW and its negative impacts
(c) the learned lessons and challenges of the CR- FLW Network as a multi-stakeholder entity.

Consultations with the CR-FLW Network members and other stakeholders allowed us to analyze how the current FLW actions drive actors and stakeholders of the Costa Rican context towards innovation for FLW prevention and reduction. Primary data (interviews and questionnaires) and secondary data (scientific and grey literature) were used to describe the background and milestones of the network, conduct the literacy analysis, and stakeholder mapping. Each subcase within the case study section follows a distinct methodology to analyze available data, but the approach as a whole showcases three examples or areas of current FLW actions in the country, as outlined in the next sections.

### 2.1. Stakeholder Analysis

This tool has been proven to support decisions and insights where an initiative is to be developed. Since the Rio Declaration of 1992, the involvement of stakeholders has been recognized as necessary to address socio-environmental aspects [27]. Because of its applicability to identify and frame active stakeholders of the Costa Rican FLW experience, it was recognized as a starting point for this study.

Many tools and methods are suitable to conduct this assessment. In our case, a self-administered online questionnaire was sent to the 25 active members of the CR-FLWNetwork using the Networks’ e-mail database. The questionnaire consisted of 14 items that included open-ended, Likert scale, and multiple-choice questions. Twenty-one (21) questionnaires were completed, and responses from individuals from the same organization were consolidated to reflect the average per institution. A total of 17 records (68% response rate) were analyzed. The answers were examined descriptively to identify, categorize and better understand the grouping and characteristics of the stakeholders belonging to the CR-FLW Network, hence part of the FLW innovation ecosystem in Costa Rica. Other secondary data allowed the investigators to complete the stakeholder map, and a graphic representation of this analysis was proposed considering the following criteria: (a) interest, (b) influence, and (c) sector. The description of the indicators for each criteria is presented in Table 1.

### 2.2. Case Study

Three subcases from the groups of stakeholders of different sectors in the food system scenario of Costa Rica illustrated three of the current key areas of action of the CR-FLW Network. These cases are characterized by having the interaction of at least two network members (identified in the previous mapping in Section 2.1); their activities have been formalized in the operative plans of their organization, documented, and supported by organizational resources; moreover, they had available data and information that was willingly shared. Each case entailed a general description, a presentation of the available data (analyzed under specific methods), and an overall assessment based on the strengths, challenges, and opportunities.

Subcase 1: Food Waste (FW) assessment in universities

Since 2018, a consortium of universities both public and private grouped under the Costa Rican Network of Sustainable Education Institutions (REDIES) has performed FW measurements [28] applying the CR-FLW Network guidelines [29] in the past, and hosting activities to raise awareness. REDIES applied an electronic survey during October 2020 to three of its members (coded as University A, B, and C) to describe their population’s behavior, knowledge, and characteristics regarding FW. The survey was applied remotely using an electronic form, since in-person activies were restricted in that moment due to the COVID-19 pandemic. It considered the three main food serving schedules (either at the university cafeteria or at home), accounting for a population of 33,402 persons distributed in the campuses of three institutions. In their assessment, REDIES calculated that the required sample was a minimum of 380 individuals (see Equation (1)); finally the study successfully collected 451 answers.

(1) $$n=\frac{\left(Z^{2}\times p\times q\right)}{d^{2}}$$

Note: *Z* = 1.96 squared for a confidence level of 95%; *p* = probability of success, or expected proportion, in this case 50% = 0.5; *q* = probability of failure, 1 − *p*, in this case 1 − 0.5 = 0.5; and *d* = precision, maximum margin of error in terms of proportion 5%.

The survey included filter questions to determine the population that belonged to the student group and the university employees’ group and the use of the institutional restaurant service. It was structured in three areas concerning the variables of interest: (a) personal information (sex, age, campus, occupation, i.e., student or employee, amount of individuals in their household), (b) FW knowledge, and (c) behavior (self-reported most frequent mealtime with FW and most wasted food). The analysis of the obtained data used the Minitab software and began with a general descriptive analysis, followed by the exploration for statistical differences among universities’ respondents through the Tukey test (α = 0.05). In addition, the relations among three variables of interest (time of service, frequency of waste, university) were analyzed using the square chi test with two categorical variables each time (α = 0.05).

Subcase 2: Redistribution actions to avoid FLW

The three members of the CR-FLW Network working on food redistribution were analyzed. The first is a direct redistribution project that works without intermediaries between the surplus source and the beneficiary organizations and is run by the National Center for Food Supply and Distribution (CENADA by its acronym in Spanish). The second is a civil society organization (CSO) called Alimentalistas that acts as an intermediary between the surplus generator entity and the target population (indirect redistribution), and third, the Costa Rican Food Bank, which is part of the Global Food Banking Network.

For the purpose of this paper, the concepts of food surplus redistribution and food donation are outlined as two different actions. Food surplus redistribution refers to safe food produced, retailed, or ready to be served (or offered) but for different reasons is not sold to or consumed by the intended customer [30] and if not recovered, it becomes waste, generally destined for landfill. In contrast, food donation refers to food with commercial value handed out for charity purposes.

The case comprised the review of institutional reports (published and unpublished), food distribution databases of each case study, and interviews with the organization’s coordinators to integrate and validate the retrieved information and further understand the main challenges of scaling up food redistribution programs in the country. For each experience within this subcase, information about their characteristics (e.g., type of program, human resources), quality of the redistributed products (e.g., food source, reasons for redistribution, food groups), and key metrics were retrieved. The sample was not representative; therefore, the results cannot be used to infer quantity or type of national-level FW. However, they represent three important avenues for reducing food waste and advocating for prevention through public engagement.

Subcase 3: Innovation call in Central America

Innovation as the response to solve challenges and needs can be obtained from triggering arrangements such as action, strategies, policies, or plans regarding a certain topic, as it supposes the exploitation and adoption of new approaches by stakeholders [17]. Therefore, this case was built on the recently celebrated Call for Innovation in the Central American Subregion, coordinated by the international platform #*SinDesperdicio* [31], where the CR-FLW Network was invited as one of the partners. Data from this subcase was developed by applying a semi-structured questionnaire consisting of open-ended questions to the leading group of Corporate and Advisory Partners of #*SinDesperdicio* and relevant partners of the Call (sponsors and incubator-accelerator firms). The questions covered aspects related to (a) the characteristics and outcomes of the call, (b) whether there was a particular focus on food loss or food waste, and (c) the overall assessment of the contesting initiatives. Answers were collected and studied to describe particular aspects or traits observed in regards to FLW innovation.

### 2.3. Literacy Analysis and Overall Assessment of the Costa Rican FLW Innovation Ecosystem

Literacy is defined as competence in a specific area of knowledge [32]. The term has been widely applied in socio-environmental studies under the premise that unless actors become literate on the matter of interest, workable and evidence-based solutions for existing challenges cannot become a reality [33,34].

In consequence, the resulting analysis can suggest the enabling context for changes and innovations to determinetely address FLW within the Costa Rican ecosystem. Therefore, it aimed to provide for the overall view of the literacy of the Costa Rican FLW stakeholders. Jointly with the stakeholder analysis, the case studies outcomes, and the identification of literacy elements, a more robust consideration of the current FLW actions resulting in solutions within the innovation ecosystem can be perceived.

Consulted sources included individuals belonging to member organizations of the CR-FLW Network, with a total of 21 answers used to analyze the knowledge, attitude, and practices of the stakeholders regarding the FLW context and actions within the current Costa Rican scenario. An additional set of five experts with profiles dedicated to international organizations and innovation agencies provided insights on the external perception of literacy of the Costa Rican FLW innovation ecosystem. Data collection tools included online forms consisting of seven closed-ended, value-scale, and open-ended questions and structured interviews for the experts. When possible, results were treated through descriptive statistics, and the experts’ comments were considered for discussion in this section.

## 3. Results

### 3.1. Stakeholder Analysis

**Figure 1:**
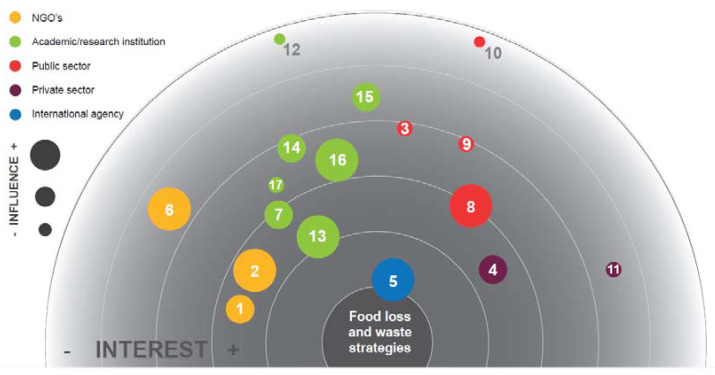
Stakeholder analysis for members of the CR-FLW network. 1. Alimentalistas, 2. Food Bank, 3. Education and Nutrition comprehensive care children’s centers (CEN CINAI), 4. Equipos Nieto SA, 5. FAO, 6. ILSI Mesoamérica, 7. National Training Institute (INA), 8. Ministry of Agriculture, 9. Ministry of Environment and Energy, 10. Ministry of Health, 11. Nutristilos (consultancy-entrepreneur). 12. State of the Nation Program. 13. The Costa Rica Institute of Technology (TEC), 14. University of Costa Rica (UCR), 15. EARTH University, 16. National University (UNA), 17. National Technical University (UTN).

Figure 1 shows the position of members within the Costa Rican FLW Network according to their self-reported level of interest (proximity to the center of the figure) and influence (size of the circle). Even though the sector is not a predictor of interest or influence within the map of stakeholders, it is important to note that all relevant sectors for FLW stakeholders are represented, including four public institutions, seven academic institutions, three non-governmental organizations, two private sector organizations, and one international agency.

Nine stakeholders work both in food loss and food waste, six only in food waste, and two exclusively in food loss. Most of the responders (70.6%) reported having worked in FLW from one to five years, 17.6% for over five years, and 11.8% for less than a year. The main activities reported included education (57%), research (48%), food service (29%), waste management (19%), food redistribution (10%), and policy (10%). The stakeholder identified with higher interest and influence was the Food and Agriculture Organization of the United Nations, which serves as the technical secretary for the CR-FLW Network, followed by TEC, which acts as coordinator (Figure 1).

Moreover, eight organizations not affiliated with the CR-FLW Network were identified as potential FLW stakeholders; however, despite being outside this paper’s scope, they are considered of interest for future engagement.

### 3.2. Subcases Description

Subcase 1: Food Waste (FW) assessment in universities

The academic sector has been a very active stakeholder in studying and foreseeing mechanisms to address FLW within their community and outreaching to other actors in society. This has resulted in testing different quantification techniques and tools that suggest that restaurants in these institutions generate 11.30% of FW (daily mean value), mostly due to leftovers from the plates of the institutional restaurant users [35]. In consequence, these studies from universities had set a base for food waste assessments and cause-inferrences for the food service in the country.

Most of the sampled population were students (81%), identified as women (67%), with an age range from 18 to 28 years old (60%), living with relatives in the same household or with study mates inside campus or nearby. A significant number of individuals (83%) were aware of the FLW concept. When asked about the impact of FLW, socioeconomic and environmental implications were reported to have high or very high correlation while nutrition outcomes were reported to have moderately-high correlation.

**Figure 2:**
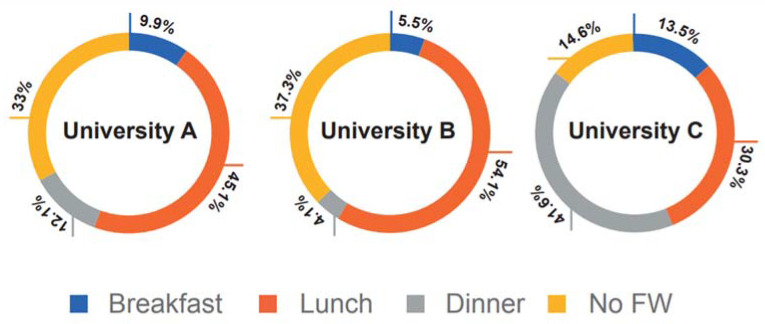
Food waste frequency per food time and respondents’ university.

Respondents were asked about the time when they tend to waste more food (either at campus or at home since the survey was applied remotely due to the COVID-19 pandemic). The individuals mentioned it was unusual to leave any food on their plates; however, when there was waste, they considered that it mostly occurred at lunchtime (Figure 2). They reported leaving less than ¼ of the food served, and from that amount, rice (37% of the time), and beans and salads (23% of the time) were the most wasted type of food. The reasons for these leftovers were the taste and the portion size, and they suggested that smaller servings might result in less wasted food. A more detailed analysis indicated there were statistical differences among the respondents from the three universities regarding the food waste report for breakfast and dinner times (*p* = 0.000), while lunchtime was statistically the same within the three institutions.

Differences among universities are explained through the positive statistical associations between particular traits of each university restaurant and (a) the time of service (breakfast, lunch, dinner), and (b) the frequency of food waste. Inferring from other answers and observations on site, knowledge and awareness are not attributable to these differences since most of the population seem to be aware of FLW. In contrast, answers in regards to taste and the time students remain on campus (some living on the premises) are particular to each university restaurant.

One strength identified when assessing this subcase is the systematic and frequent monitoring of FLW carried out by this group [35,36]. This presents an opportunity for both supporting decisions at the administrative level and raising awareness in such varied groups of individuals present in universities (students from different regions of the country and diverse career paths, where FLW sensitization can be included). Challenges have been identified in regards to the self-reporting mechanism used, and the different levels of awareness declared in the survey that may influence the understanding of the implications of FLW.

Subcase 2: Redistribution actions to avoid FLW

Three stakeholders within the CR-FLW Network include food redistribution among their activities (Table 2) and impact different actors in these context.

#### 3.2.1. Organization Profiles

CENADA is a governmental-run wholesale market for the supply of perishable products (vegetable products, fresh, dry, and preserved foods, flowers, white and red meats, eggs, and dairy products) for their subsequent distribution to retail markets. It started its food redistribution efforts in 2019 under the premise of streamlining the collection of products by vulnerable populations from concessionaires located in the market. This governmental program (GP) follows a direct-distribution methodology, while both the Food Bank (FB) and the Civil Society Organization (CSO) work through indirect redistribution. Unlike the last two, the aim of the GP was not conceived for food waste reduction purposes. The project’s scope is threefold: first, organize the market and improve its public image; second, decrease the volume of organic waste produced within the facilities; and third, improve the food security of beneficiaries by implementing food safety protocols. Redistribution is not mandatory in the wholesale market; the willingness to redirect the surplus depends on the judgment of each concessionary. Food is weighed and distributed proportionally to the number of people served by each organization, collected daily, and not stored in the facilities.

The Food Bank (FB) is a non-governmental organization representing Costa Rica’s biggest food donation and food surplus distribution agency. Their operations and logistics are similar to other Global Food Banking Network [37] members and have two branch offices in the country. It uses a warehouse model and supplies food to intermediaries like charities and other NGOs.

The CSO called Alimentalistas was founded in 2016. The name Alimentalistas comes from Environmentalist and Food (*Ambientalista* and *Alimento*, in Spanish). This CSO seeks to reduce food waste through prevention, redistribution, and awareness. Prevention strategies include advocacy and education campaigns (e.g., workplace, household-level, schools, and universities), social media outreach, and technical support to the food business and other stakeholders. At the CSO, food rescue events are seen as education opportunities among volunteers and the surplus source. Food rescue logistics rely on small loads and short supply chains, and food is packed in biodegradable containers and placed in reusable plastic boxes. Volunteers use their own vehicles and deliver within 1–10 km of the surplus source; therefore, this model does not require storage facilities. Data is collected by trained volunteers using electronic kitchen scales (5000 g × 1 g) for food at events and restaurants and a produce scale (20 kg × 50 g) for fruits and vegetables at farmer’s markets. When food is weighed, non-edible parts (bones, peels, etc.) are included and food is handled with the least possible manipulation. Records of each type of food item and weight are tabulated into an Excel spreadsheet together with information to ensure traceability (date, location, photographic evidence, name of the volunteers, and name of the beneficiary organization).

#### 3.2.2. Type of Redistributed Food

Both the FB and the GP receive food surplus and food donations, while the CSO does not distribute food donations. The CSO classifies food according to the main ingredient in 17 food groups adapted from the Food Composition Table of the Institute of Nutrition of Central America and Panama [38]. All meat categories are grouped into one, and eggs, shellfish, and fish are omitted since they are not rescued due to food safety concerns. Between January 2019 and December 2020, the CSO rescued 8164 kg, with the main food groups consisting of fruits and vegetables (33%), bread, tortillas, and pastries (28%), meats (12%), and grains and legumes (9%). It has nutrition protocols to improve the quality of the food delivered. For example, it encourages the distribution of fruits and vegetables, limits confectionery and desserts, and prohibits sodas, energy drinks, and packaged products with sugar or fat as the first ingredient. In contrast, the FB doest not have formal nutrition policies, but there is an ongoing effort to increase the number of fruits and vegetables the FB receives (currently around 2%). It receives both food and non-food items (cleaning supplies, toiletries, clothing, among others), and it is estimated that about 65% of the distributed products are food, and in the last fiscal year it handled around 3,690,000 kg (October 2019–December 2020). The GP only distributes fruits and vegetables, and it has rescued 234,000 kg between September 2020 and May 2021.

#### 3.2.3. Reported Challenges

Food donation and redistribution initiatives are diverse in their organizational structure and are independent of each analyzed organization. Nevertheless, they share common challenges as the country requires policy instruments to support and advance these actions. The lack of legal liability for food donors makes stakeholders reluctant to redistribute their surplus and, on many occasions, prefer to discard it to avoid being legally pursued in case of food safety issues. The financial burden incurred by the organization is also one of the main barriers to better their operations. For these initiatives, the inconsistent and insufficient funding results in limited investment in infrastructure for storage, handling, and transportation of food and heavy dependency on volunteers. The limited staff available and the volunteer’s variability across the year hinder operational capacity and the feasibility of responding to seasonal spikes. Attracting and retaining volunteers represents a challenge since it requires robust FWL sensibilization strategies among the general public, which is a resource-intensive task.

Subcase 3: Innovation contest in Central America hosted in Costa Rica

FLW reduction in Latin America has been approached in different manners. One initiative is the regional platform *#SinDesperdicio*, which integrates international organizations and private sector partners, aiming to promote innovation, public policy, knowledge, and changes in behavior to address FLW [31]. #*SinDesperdicio* has led four innovation calls. One, in particular, was located in the Central American subregion, considering Costa Rica as the headquarters, due to the existing environment regarding FLW reduction in the country. The Call consisted of an open innovation tender targeting FLW and therefore considering technological, product, social, organizational, or marketing innovations applicable to different stages of the food supply chain. Once the contest was launched, several webinars motivated and communicated the terms, conditions and context under which innovative solutions were to be foreseen. The ideas were submitted online and pre-evaluated by a group of experts recommended by different partners of #*SinDesperdicio*. A total of 135 applications were received, resulting in 12 finalists who went through a Virtual Bootcamp and a Final Virtual Pitch. Twenty-five percent of the finalists were Costa Rican, some of them related to the stakeholders of the CR-FLW Network, acting as innovators. A group of jurors assessed the initiatives to select three winners who earned USD10,000 each as seed capital and a specialized acceleration companionship. One of the selected innovations belonged to a Costa Rican participant who proposed the use of beeswax for sustainable and reusable packaging that would allow better conservation of food products.

Coordinators of this activity indicated that neither the Call nor the participants peresented a srict focus on food loss or food waste. However, according to experts and partners behind this innovation Call, there was a slight emphasis on addressing efficiency at the early stages of the supply chain (losses) since other stages entailing food waste would have multidimensional causes. This latter stage was considered as something difficult to address, especially in early innovation processes. Nevertheless, many participants focused on waste as a whole concept and aimed at treating it and not necessarily preventing FLW.

This subcase study suggests that the experience of developing an innovation contest was positive, demonstrated by the support shown by regional and Costa Rican partners and the number of applications received by candidates. The respondents highlighted the educational, science, and research background observed in many Costa Rican contestants, which helped generate more comprehensive, contextualized, and well-aligned proposals. However, experts also considered some potential solutions to be too specific and detected a relevant number of “end-of-pipe” alternatives, which expresses a limitation in the systemic scope needed to address FLW.

### 3.3. Literacy Analysis

The literacy analysis was employed to identify the enabling set of elements related to knowledge, attitude, and practices towards FLW reduction, as seen in Table 3. Most of the respondents (52%) claim to have a wide or at least moderate knowledge of FLW. A relevant group of answers also indicates a positive attitude towards tackling FLW (over 60%), either by receiving organizational support, considering FLW as key in their activity, or considering they put efforts into promoting innovation to avoid or reduce FLW. Finally, most of the respondents indicate having practices that enable FLW prevention or reduction (positive assessment among 57% to 66% of respondents), such as a plan to define actions in this matter and specific mechanisms or tools to do that.

When addressing barriers or limitations, the most frequent constraint according to the consulted sources were budget and public and policy support to allow FLW actions to fluently move forward. These aspects also add to the barriers detected by external experts who observe an overall lack of investments in research and innovation in the country. Literature sources and reports consistently indicate that one relevant challenge in the country relates to R&D&I investment, particularly agri-food sector investments [39,40], somehow linked as well to some of the hindering factors mentioned in other literature [11]. Specialized attention or support to productive actors in an articulated manner is another limitation, which can be potentially surpassed due to multi-stakeholder approaches such as as the CR-FLW Network. This latter action can allow an improved potential to apply approaches based on knowledge, bioeconomy, and circular economy, including the interaction with not only technical actors but also from incubators, accelerators, media, and others already in the FLW Network who can play a key role.

Nonetheless, even with limitations, the stakeholders also indicate that when budgets have been allocated, they have played a crucial role as enablers to promote FLW actions, together with knowledge and the policy background that exist in the country, which even when not FLW-specific, undertakes the topic and can trigger actions, especially in the institutional side. Other enabling factors observed by external experts suggest the relevant role of existing legislation and policies that already support the need to work on FLW reduction, such as the Costa Rican waste management framework, present in the country since 2010. Moreover, the experts claimed that the already existing public-private liaison born from the CR-FLW Network and academia seemed to provide a paramount opportunity to accelerate the process towards increased actions to reduce FLW.

## 4. Discussion

### 4.1. Highlights from the Case Study

The case study helped illustrate the current scenario of FLW actions in Costa Rica, coming from mapped stakeholders. In this sense, the universities’ subcase suggests how monitoring and inference of causes in university populations can aid future decision-making and awareness-raising. FLW interventions are present in many studies from educational institutions [36,41,42] around the world. For instance, appreciations from the interviewees are supported by literature when considering the multidimensional reasons for their FLW: the size of servings, type of food that is wasted, and meal schedules play a significant role in the FLW behavior [42,43]. Understanding the causes of FLW from these individuals is relevant for the university stakeholders since it can provide valuable indicators for a more comprehensive waste management strategy aligned with national efforts to reduce GHG emissions and manage waste [36]. In addition, universities can be perceived as small towns within larger cities [44]; therefore, creating awareness in such socioeconomic and origin-diverse populations can allow for a broader impact in the surrounding context. In parallel, addressing FLW at universities can help validate strategies to be later applied in other similar contexts. This case study shows that there is already existing FLW awareness in students, presenting an opportunity to introduce the topic in further professional environments. As interviewees from this subcase expressed it, FLW has significant relations to environmental impacts and nutrition; therefore, actions generated from pilot cases like the universities could potentially impact FLW actual metrics and other already known socioeconomic or environmental indicators derived from FLW.

Differences in concepts (redistribution vs. donation) and mechanisms (solidarity and charitable approaches, corporate responsibility, or legislation as the Italian Law n. 166/2016 or the French Law Loi n° 2016-138” to ban FLW) are often encountered when addressing food donation/redistribution as a FLW-related strategy. These conflicting definitions makes it difficult to implement strategies with an effective appropriation of concepts and actions by users [44,45,46]. The CR-FLW Network vision acknowledges that food redistribution is not the primary solution to tackle food waste nor food security; nonetheless, it is recognized as an important safety net to connect available surplus food with beneficiaries [47] since actions in this dimension result in actual avoidance of FLW as food finally arrives to someone who will make good use of it. Even though only a fraction of the food surplus is redistributed, these ventures represent an opportunity to connect consumers with the socio-ecological footprint of food waste. Engaging with volunteers can have a trickle-down effect on individual behavior as they participate in redistribution activities. Well-informed consumers can highlight the need and push for actions to incentivize food waste prevention and management options in their food environment [48]. In parallel, the case study also presents part of the current challenges already expressed in the Atlas of Food Donation [49]. Potential improvement in policies, tax incentives, and education can enable donation as an alternative for FLW reduction to a certain degree. In addition, since food redistribution includes such a diverse set of actors across the supply chain, these efforts and partnerships could pave the way for more comprehensive food waste prevention strategies through collaborative multi-stakeholder engagement.

Finally, the case study on the *#SinDesperdicioCentroamérica* innovation call suggests that the FLW topic is somehow present in innovators and potential entrepreneurs in Central American countries, and related stakeholders since a set of different institutions and organizations joined efforts to promote FLW reduction ideas. FAO presented at the beginning of the COVID-19 pandemic a set of three potential areas of innovation to prevent FLW [19]. Many of these trends were present in the *#SinDesperdicioCentroamérica* Call, such as apps to maximize food sale [20], donation or distribution, or equipment and technology applications [21]. However, these examples are motivated mainly by micro and meso level causes of FLW that need to be embedded into more systemic approaches to tackle FLW. Experts from this case study supported this notion, suggesting that actions related to the CR-FLW Network (webinars, mentoring, research outcomes, and examples from the other two case studies) are beginning to trigger a shift in the FLW approach in the national context, with a deep understanding of comprehensive innovation approaches.

### 4.2. Status of FLW Actions in Costa Rica, Learned Lessons and Challenges

The stakeholder analysis portrays the diversity of members in the Costa Rican FLW scenario, which is helpful to promote interactions, complementary actions, and capabilities that can positively affect FLW activities, as suggested by many sources [12,13,14,15,16]. The case studies show the advantages of multi-stakeholder involvement in addressing FLW, data gaps, and sharing experiences. On the one hand, trends and behaviors in the university foodservice subsector began to be mapped when several entities came together and agreed to work within the same methodological frameworks. This not only allows us to conduct comparisons between institutions but also creates collaborative spaces to design comprehensive solutions. FLW estimation and quantification is crucial when monitoring the advancement towards SDG 12.3. With the guidelines validated in the universities and the collection of inferred FLW causes and behaviors, other types of restaurants (from hotels, small businesses, institutional services) can begin collecting their data and support sector monitoring and intervention.

Innovation calls like *#SinDesperdicioCentroamerica* exemplify the need for several actors already present on local and international platforms to work in a collaborative manner. By adding local stakeholders like the CR-FLW Network, Ministries, and innovation experts who provided feedback and training to organizers and participants, the contest addressed FLW reduction and prevention along the food supply chain. The Call itself also represented an opportunity to raise awareness among the general public.

Moreover, innovations operating in the Costa Rican context through donation and redistribution of food surpluses set the scene for FLW prevention and reduction, and alternative food supply chains. Actions of the Food Bank, Alimentalistas, or CENADA are only possible due to alliances with food producers and distributors, volunteers, organizations, and beneficiaries that integrate a new value chain. In addition, these CR-FLW Members interact to provide advice and exchange experiences and good practices among them.

Even though strong and long-term partnerships have enabled FLW actions since 2014 through the CR-FLW Network, scaling up in Costa Rica is seen as a challenge. The lack of financing streams to support the operations, the heavy dependency on volunteers, and the lack of incentives for private companies to do volunteer work or invest in innovation and research are barriers to expand operations in scenarios like the ones related to food donation and redistribution.

Moreover, a labeling system that does not differentiate quality and safety indicators, inadequate tax benefits and penalties, and the lack of legal liability for food donors further hinders an innovation ecosystem within the food redistribution sector [49]. These nuances underline the shortcomings of traceability, quantification, and overall surplus management; however, a robust framework and platform for food management can propell new value chains and businesses to transform surplus into innovative products, a venue that is still incipient in the country.

Knowledge is identified as a critical element for the innovation system, which is why the presence of Network actors focused on education and awareness could facilitate capacity building and strengthen the availability of information to promote innovative approaches to FLW. According to our findings, the studied population acknowledges FLW as an issue and recognizes the high social-economic and environmental impact it represents. In addition, they claim to have prevention and reduction practices with specific mechanisms and tools to carry out their actions, so knowledge is not recognized as a limitation in the system. In contrast, the availability of financial resources is a constraint. Budget obstacles limit the progress and the capacity to scale up actions in the country, a situation already stated in many national studies [39,40]. Even though the stakeholders refer to budget limitations, the increased interest, design, and implementation of national policies [50,51] driven by the global sustainability agenda could advance budget distribution and help bridge the gaps within the innovation system.

The pursuit of systemic change could be considered as a potential outcome of cross-sector partnerships [52]. In complex challenges such as FLW reduction, the multi-stakeholder and collaborative platforms with a common purpose like the CR-FLW Network represent a venue to promote systemic change. The coordination of system-level changes with micro-level ones through the network could promote a bottom-up approach to policy. This fundamental change in policy driven by the network’s members can help address the power dynamics in the supply chain and assure an equitable distribution of responsibility among actors when designing solutions. Innovative approaches to address FLW can promote changes in production and consumption patterns once they trigger knowledge, interest, actions and needs that can be later undertaken by sectoral or national policies. For instance, composting has not been seen as a potential agribusiness in the past; nowadays, a national policy for composting promotes the implementation of technology, improved techniques, and economic feasibility of these approaches, beginning with a concept of FLW reduction as part of the preferred hierarchy of waste management [53].

Many challenges need to be addressed to achieve a more robust and permanent collaboration that allows a comprehensive inclusion of FLW in the national agenda. Potentially, the interventions at university foodservice and the awareness of their users, the diverting of food surplus from landfills by dedicated organizations, and the new business models presented in the innovation calls could result not only in FLW reduction but also address the negative externalities such as GHG emissions, waste of resources, land use impacts from landfills and waste treatment alternatives, food insecurity and related costs to FLW disposal.

## 5. Conclusions

A growing number of initiatives aiming at the reduction of food waste have emerged in the country. This array of interventions are increasingly recognized to have a pivotal role in reducing the negative impacts of FLW in the food system by prioritizing source reduction and recovery strategies and triggering production and consumption patterns in the food system.

Through the work of the CR-FLW Network, the Costa Rican case study highlights the advantages of multi-stakeholder collaboration to bring public, private, academia, and civil society actors closer together and advance actions aimed at reducing FLW. The authors believe that this model could be valuable for other countries in the region as they work towards achieving the Sustainable Development Goals. Nonetheless, while the network has the potential to trigger comprehensive policies and sound strategies, further political engagement is needed to promote an integrated innovation ecosystem that expands across all sectors.

Finally, the work that has been carried out by the Costa Rican FLW Network and its members, such as REDIES, Alimentalitas, the Costa Rican Food Bank, CENADA, as well as its interrelation with other regional platform and national actors can help promote and sensitize the different sectors of the population to prevent and reduce FLW.

## Figures and Tables

**Table 1:** Criteria and indicators used to conduct the stakeholder analysis.

Criteria	Description and Indicators
Interest	Refers to the priority given by stakeholders to food loss and waste (FLW) within their organization, the impact that FLW has in their organization, and their participation in the Costa Rican Food Loss and Waste Reduction Network (CR-FLW Network). It was calculated through the sum of self-reported score related to the priority given to FLW within their organization (5-point scale), the impact that FLW has in their organization (5-point scale), and the percentage of CR-FLW Network meetings attended between November 2014 and April 2021 (1 = <20%, 2 = 20–39%, 3 = 40–59%, 4 = 60–79%, 5 = 80–100%) All items are assumed to have equal weight when calculating the added score per member and plotted on the graph according to the given scale.
Influence	Considers if the stakeholder collaborates with other members on FLW projects, if they have organizational support for FLW activities and if the organization has an explicit plan to address FLW. It was calculated through the sum of self-reported scores related to the collaborative FLW work the organization develops with others (5-point scale), the organizational support to the FLW topic (5-point scale,) and if the organization has an explicit plan to address the FLW topic (5-point scale). All items are assumed to have equal weight when calculating the added score and plotted on the graph according to the given scale.
Sector	The mapping considered four main sectors and clustered the stakeholders in (a) public, (b) private, non-governmental organizations (NGO) or civil society, (c) education and academic, and (d)international organization.

**Table 2:** Characteristics of the food redistribution actors within the CR-FLW Network.

	Direct Redistribution	Indirect Redistribution	
Name	CENADA	Alimentalistas	Food Bank- Costa Rica
Type	Governmental-run project (GP)	Civil society organization (CSO)	Non-governmental organization (codified in this document as FB)
Food source	Wholesale market of perishable products	Diverse sources of food (e.g., producers, events, restaurants, catering services, farmers markets)	Supermarkets, food industries, commercial distribution companies, producers
Reasons for food distribution	- Food donation - Food surplus (aesthetic standards and seasonal variations on demand)	- Food surplus (overproduction, difficulty in anticipating customer numbers in catering services, aesthetic standards, seasonal variations on demand)	- Food donation - Food surplus (overproduction, aesthetic standards, mislabeled products, expiration dates)
Human resources	Volunteers Staff with partial dedication	Volunteers	Volunteers Dedicated staff
Key metrics	- Volume of food distributed (kg) - Number of beneficiary organizations	- Civil society engagement: number of enrolled volunteers, number of active volunteers - Education and advocacy activities organized - Number of food rescue events - Volume of food rescued (kg)	- Volume of food distributed (kg) - Number of charities served - Number of volunteers - Monetary donations - Number of donors

**Table 3:** Knowledge, attitudes, and practices from stakeholders to address FLW.

	Completely Disagree	Disagree	Neither Disagree Nor Agree	Agree	Completely Agree	Total
Knowledge:						
There is widespread knowledge about FLW in the organization	10%	5%	33%	23%	29%	100%
Attitude:						
FLW is a priority within their actions	10%	10%	19%	37%	24%	100%
There is organizational support for FLW actions	10%	0%	24%	28%	38%	100%
Explicit actions to promote FLW innovation exist	10%	0%	24%	28%	38%	100%
Practices:						
There is a plan to address FLW systematically	10%	14%	19%	33%	24%	100%
Specific tools, methods, and actions to tackle FLW are identified and used	10%	10%	19%	19%	43%	100%

## Data Availability

The data presented in this study are available on request from the corresponding author.
